# Disease relapse rate in children with autoimmune rheumatic diseases after COVID-19 infection and vaccination

**DOI:** 10.1186/s12969-023-00829-4

**Published:** 2023-05-19

**Authors:** Tjaša Šinkovec Savšek, Mojca Zajc Avramovič, Tadej Avčin, Miša Korva, Tatjana Avšič Županc, Nataša Toplak

**Affiliations:** 1 Department of Allergology, Rheumatology and Clinical Immunology, University Children’s Hospital, University Medical Centre Ljubljana, Bohoričeva ulica 20, Ljubljana, 1000 Slovenia; 2 Faculty of Medicine, University of Ljubljana, Ljubljana, Slovenia; 3 Institute of Microbiology and Immunology, Faculty of Medicine, University of Ljubljana, Ljubljana, Slovenia

**Keywords:** COVID-19, SARS-CoV-2 infection, Vaccination, Paediatric autoimmune rheumatic diseases, Relapse rate

## Abstract

**Background:**

Paediatric patients with autoimmune rheumatic diseases (pARD) are often immunocompromised because of the disease and/or the therapy they receive. At the beginning of COVID-19 pandemic there was a great concern about the possibility of severe SARS-CoV-2 infection in these patients. The best method of protection is vaccination, so as soon as vaccine was licenced, we aimed to vaccinate them. Data on disease relapse rate after COVID-19 infection and vaccination are scarce, but they play important role in everyday clinical decisions.

**Methods:**

The aim of this study was to determine the relapse rate of autoimmune rheumatic disease (ARD) after COVID-19 infection and vaccination. Data on demographic, diagnosis, disease activity, therapy, clinical presentation of the infection and serology were collected from pARD who had COVID-19 and from pARD who were vaccinated against COVID-19, from March 2020 to April 2022. All vaccinated patients received two doses of the BNT162b2 BioNTech vaccine, on average, 3.7 (S.D.=1.4) weeks apart. Activity of the ARD was followed prospectively. Relapse was defined as a worsening of the ARD in a time frame of 8 weeks after infection or vaccination. For statistical analysis, Fisher’s exact test and Mann-Whitney U test were used.

**Results:**

We collected data from 115 pARD, which we divided into two groups. We included 92 pARD after infection and 47 after vaccination, with 24 in both groups (they were infected before/after vaccination). In 92 pARD we registered 103 SARS-CoV-2 infections. Infection was asymptomatic in 14%, mild in 67% and moderate in 18%, 1% required hospitalization; 10% had a relapse of ARD after infection and 6% after vaccination. There was a trend towards higher disease relapse rate after infection compared to vaccination, but the difference was not statistically significant (p = 0.76). No statistically significant difference was detected in the relapse rate depending on the clinical presentation of the infection (p = 0.25) or the severity of the clinical presentation of COVID-19 between vaccinated and unvaccinated pARD (p = 0.31).

**Conclusions:**

There is a trend towards a higher relapse rate in pARD after infection compared to vaccination and connection between the severity of COVID-19 and vaccination status is plausible. Our results were, however, not statistically significant.

**Supplementary Information:**

The online version contains supplementary material available at 10.1186/s12969-023-00829-4.

## Background

Coronavirus disease 2019 (COVID-19) is a highly contagious disease caused by severe acute respiratory syndrome coronavirus 2 (SARS-CoV-2) [1, 2]. Infected children usually suffer from systemic and respiratory symptoms such as fever, cough, sore throat, runny nose, sneezing, and nasal congestion [2]. The clinical presentation of COVID-19 in children is milder and the outcome favourable compared to adults. In children with COVID-19 who required hospitalization, the mortality rate was approximately 0.2% [2].

Because patients with autoimmune rheumatic diseases (ARD) have a dysregulated immune system there was a concern that they could be more susceptible to COVID-19 and have a more severe clinical presentation [3]. Studies on adult patients with ARD did not prove this, as clinical features of SARS-CoV-2 infection, the need for hospitalization, and the mortality rate were similar in patients with and without ARD [4]. In Atlanta, USA, a study was done among paediatric patients with ARD (pARD) infected with SARS-CoV-2. The researchers concluded that a worse clinical presentation and increased odds for hospitalization were associated with African American race, pre-existing cardiovascular disease, active ARD at the time of infection, and severe immunosuppression [5]. Another study was performed in Washington, DC, USA, where underlying medical conditions were reported to play an important role in the severity of COVID-19 in children. The most common underlying diagnosis was asthma, but diabetes, obesity, neurologic, hematologic, and oncologic conditions were also recorded [6].

The EULAR study, published in March 2022, included 607 children and adolescents with ARD from 25 different countries. Results showed that 43 (7%) patients required hospitalization and among these three patients died, indicating that the majority were not hospitalized and required no intervention [7].

Knowing that most pARD were not at risk for a more challenging course of COVID-19 was relieving but questions about the impact of COVID-19 on ARD remained unanswered. A German study showed that SARS-CoV-2 infection caused flares in children with juvenile idiopathic arthritis (JIA). Relapses were seen in 7 out of 13 (54%) JIA patients who had a positive serology for SARS-CoV-2 [8].

The first vaccine approved for adolescents aged 12–18 years was the BNT162b2 Comirnaty (Pfizer-BioNTech), based on the delivery of messenger RNA encoding the SARS-CoV-2 spike glycoprotein [9, 10]. A study done in Israel enrolled 91 pARD and healthy controls who were vaccinated with two doses of the BNT162b2 Comirnaty vaccine. It was shown that the vaccine is safe and effective. Changes in immunomodulatory medications were required in eight patients after the first and in four patients after the second dose of vaccine [11].

## Methods

The aim of the study was to investigate the relapse rate of ARD in pARD after SARS-CoV-2 infection and after vaccination against COVID-19. It was a single-centre study conducted between March 2020 and April 2022, at the University Children’s Hospital in Ljubljana, Slovenia.

We followed pARD after infection and vaccination prospectively. Data on COVID-19 infection were collected at regular visits in the rheumatology outpatient clinic.

### Study population

The study population included children (ages 2–12 years), adolescents (ages 12–18 years), and young adults (ages 18–23 years) with ARD including JIA, systemic lupus erythematosus (SLE), systemic vasculitis, idiopathic uveitis, systemic or juvenile localized scleroderma (jLS), juvenile dermatomyositis (jDM) and chronic recurrent multifocal osteomyelitis (CRMO). Other, less common diagnoses included rheumatic fever, cryopyrin-associated periodic syndrome (CAPS) and undifferentiated connective tissue disease (UCTD). All patients were diagnosed according to the valid criteria for their respective disease [12–18].

Patients were divided into two groups based on the criteria they met:

- Group 1 (pARD after infection): laboratory-confirmed SARS-CoV-2 infection with a real-time RT-PCR test or a rapid antigen test or positive serology for IgG anti-SARS-CoV-2 antibodies in pARD with a confirmed contact with SARS-CoV-2.
- Group 2 (pARD after vaccination): patients who received at least two doses of the BNT162b2 Comirnaty vaccine in a 3–9 weeks’ time span.

### Serology for IgG anti-sars-cov-2 antibodies

In addition to real-time reverse transcription-polymerase chain reaction (RT-PCR) assays used to confirm acute infections with SARS-CoV-2, establishing the antibody immune response is very important for the retrospective determination of COVID-19 in asymptomatic patients with a confirmed contact with SARS-CoV-2. For this purpose, measurement of total anti-SARS-CoV-2 IgG antibodies is used [19]. In the present study anti-SARS-CoV-2 IgG antibodies were measured with Anti-SARS-CoV-2 ELISA (IgG) EUROIMMUN, Lübeck, Germany, according to the manufacturer’s instructions and as previously described [20]. A sample is defined as positive for the presence of anti-SARS-CoV-2 IgG antibodies when the calculated value is greater than or equal to 1.1. When the calculated value is less than 0.8, the sample is defined as negative. According to the manufacturer’s instructions, samples with values between 0.8 and 1.1 are defined as “threshold values” [20, 21].

### Severity of clinical presentation of SARS-cov-2 infection

Patients from Group 1 (pARD after infection) were divided in four subcategories based on the symptoms:

- Asymptomatic infection: patient did not report any symptoms.
- Mild clinical presentation: patient reported a loss of smell and/or taste, fever of 38 °C or less for up to two days, mild headache and/or cough with no other symptoms.
- Moderate clinical presentation: patient reported fever lasting more than two days or above 38 °C, myalgia, and/or other symptoms not included in the previous subcategory.
- Hospitalization: because of SARS-CoV-2 infection, patient required hospitalization.

### Relapse of autoimmune rheumatic disease

To assess whether a patient had a relapse of ARD after COVID-19 or vaccination against COVID-19, four categories were defined:

- No relapse.
- Mild relapse of ARD: patient suffered a clinical worsening of ARD however no treatment adjustment was required.
- Moderate relapse of ARD: patient suffered a clinical worsening of ARD that required a treatment adjustment.
- Severe relapse of ARD: patient suffered a clinical worsening of ARD which required a treatment adjustment in the hospital (hospitalization).

For a clinical worsening of ARD to be considered a result of COVID-19 or vaccination against COVID-19, the relapse had to occur up to 8 weeks after infection or vaccination. Potential relapse was assessed by medical history, clinical examination, laboratory results (when available), and a VAS numerical grade obtained from caregivers and patients. In patients with JIA, juvenile arthritis disease activity score 10 (JADAS10) before and after infection or vaccination was used as an objective measure [22].

### Statistical analysis

Statistical analysis was performed using the IBM SPSS program (version 22). Fisher’s exact test and Mann-Whitney U test were used to compare two groups of categorical variables. All tests used were two-tailed and a *P*-value ≤ 0.05 was considered statistically significant.

The study was approved by the Medical Ethics Committee of the Republic of Slovenia. Written informed consent was obtained from parents or caregivers and from patients, if older than 15 years.

## Results

### Study group

We collected data for 115 pARD and divided them into two groups. We included 92 pARD after SARS-CoV-2 infection (Group 1) and 47 pARD after vaccination with two doses of the BNT162b2 Comirnaty vaccine (Group 2). Of those, 24 patients were included in both groups as they were infected with SARS-CoV-2 before or after vaccination. Study group characteristics are summarised in Table 1. The mean age was 13.4 (S.D.=4.1) years in Group 1, and 15.9 (S.D.=2.4) years in Group 2; 73% of patients were female in Group 1, and 64% in Group 2. The most common diagnosis was JIA, with 81% in Group 1 and 86% in Group 2. Other diagnoses such as SLE, vasculitis, uveitis, jDM, CRMO, jLS, rheumatic fever, CAPS, and UCTD were less common (Table 1).

In 47 pARD, a total of 94 vaccinations were performed. On average, patients received the second dose of vaccine 3.7 (S.D.=1.4) weeks after the first dose.

Altogether, we registered 103 SARS-CoV-2 infections. In 94 (91%) cases, a nasal swab was collected and either a real time RT-PCR or rapid antigen test for SARS-CoV-2 was performed. The results were positive in 65 (69%) and negative in 29 (31%) cases. Of the patients with a negative SARS-CoV-2 direct test, 20 (69%) reported contact with a SARS-CoV-2 positive person (positive contact) and had typical clinical symptoms of COVID-19. The other 9 (31%) also reported positive contact but did not recall any symptoms of COVID-19. In these patients, SARS-CoV-2 infection was confirmed retrospectively with serology.

SARS-CoV-2 testing data were not available for 9 (9%) cases. Seven (78%) reported positive contact and typical clinical symptoms of COVID-19. The other two (22%) also reported positive contact but no clinical symptoms, thus SARS-CoV-2 infection was confirmed retrospectively by positive IgG anti-SARS-CoV-2 antibodies.

**Table 1:** Patients’ basic characteristics

Characteristics	Group 1^*a*^ (*n* = 92)	Group 2^*b*^ (*n* = 47)
Mean age (S.D.) in years	13.4 (4.1)	15.9 (2.4)
≥ 12 years, *n* (%)	61 (66)	46 (98)
Females, *n* (%)	67 (73)	30 (64)
Diagnosis		
JIA^*c*^, *n* (%)	74 (81)	40 (86)
SLE^*d*^ and vasculitis, *n* (%)	3 (3)	0 (0)
Uveitis, *n* (%)	3 (3)	2 (4)
jDM^*e*^, *n* (%)	2 (2)	0 (0)
CRMO^*f*^, *n* (%)	2 (2)	2 (4)
jLS^*g*^, *n* (%)	3 (3)	1 (2)
jSS^*h*^, *n (%)*	0 (0)	1 (2)
Other^*i*^, *n* (%)	5 (6)	1 (2)

^a^ Group 1 – pARD after infection, ^b^ Group 2 – pARD after vaccination, ^c^ JIA – juvenile idiopathic arthritis, ^d^ SLE – systemic lupus erythematosus, ^e^ jDM – juvenile dermatomyositis, ^f^ CRMO – chronic recurrent multifocal osteomyelitis, ^g^ jLS – juvenile localised scleroderma, ^h^ jSS – juvenile systemic scleroderma, ^i^ Other – cryopyrin-associated periodic syndrome, rheumatic fever and undifferentiated connective tissue disease

In Group 1, 69 (75%) patients were receiving immunomodulatory medications (Table 2). Twenty-three (25%) were using conventional synthetic disease-modifying anti-rheumatic drugs (csDMARDs), 30 (33%) were using biologic DMARDs (bDMARDs), and 16 (17%) were using a combination of csDMARDs and bDMARDs. Fifty-one (74%) were receiving one, 16 (24%) were receiving two, one (1%) was receiving three, and one (1%) was receiving four medications. Twenty-three (25%) patients were not taking any medications at the time of SARS-CoV-2 infection.

In Group 2, 32 (68%) patients were receiving immunomodulatory medications (Table 2). Ten (21%) were using csDMARDs, 10 (21%) were using bDMARDs, and 12 (26%) were using a combination of csDMARDs and bDMARDs. Seventeen (53%) were receiving one and 15 (47%) were receiving two medications. Fifteen (32%) patients were not taking any medications at the time of vaccination against COVID-19.

The mean JADAS10 score in patients with JIA before vaccination was 1.1 (S.D.=1.75). Three patients with JIA had a JADAS10 score ≥ 5, their scores were 5, 6, and 7, respectively. Before vaccination, patients with other diagnoses were mostly in remission. The exception was one patient with jLS who had active lesions, but she was vaccinated before her first visit to the clinic.

**Table 2:** Medications among patients

Medication	Group 1^*a*^ (*n* = 90)	Group 2^*b*^ (*n = 47*)
NSAIDs^*c*^, *n* (%)	1 (1)	0 (0)
GCS^*d*^, *n* (%)	5 (6)	0 (0)
csDMARDs^*e*^		
Hydroxychloroquine, *n* (%)	3 (3)	1 (2)
Sulfasalazine, *n* (%)	1 (1)	2 (4)
Methotrexat, *n* (%)	23 (25)	12 (26)
Leflunomide, *n* (%)	3 (3)	4 (9)
Mycophenolate mofetil, *n* (%)	5 (6)	3 (7)
Cyclosporine, *n* (%)	1 (1)	2 (4)
Azathioprine, *n* (%)	1 (1)	0 (0)
bDMARDs^*f*^		
TNFα inhibitors, *n* (%)	37 (41)	19 (40)
IL-6 inhibitors, *n* (%)	2 (2)	2 (4)
IL-1 inhibitors, *n* (%)	3 (5)	1 (2)
Rituximab, *n* (%)	2 (2)	0 (0)
Abatacept, *n* (%)	2 (2)	0 (0)
Targeted synthetic DMARDs^*g*^		
Baricitinib, *n* (%)	1 (1)	1 (2)

^a^ Group 1 – pARD after infection, ^b^ Group 2 – pARD after vaccination, ^c^ NSAIDs – nonsteroidal anti-inflammatory drugs,

^d^ GCS – glucocorticosteroids, ^e^ csDMARDs – conventional syntetic disease-modifying anti-rheumatic drugs

^f^ bDMARDs – biologic disease-modifying anti-rheumatic drugs, ^g^ Targeted synthetic DMARDs – targeted synthetic disease-modifying anti-rheumatic drugs

### Clinical presentation of COVID-19

Altogether we recorded 103 infections in 92 patients, of whom 11 (12%) got the infection twice. The mean time between the first and the second infection was 45.6 (S.D.=13.1) weeks. In 14 (14%) cases, the infection was asymptomatic, 69 (67%) had a mild and 19 (18%) a moderate clinical presentation. One (1%) patient required hospitalization.

The first pARD in Slovenia were vaccinated at the end of March 2021. A subgroup of patients who had COVID-19 between April 2021 and April 2022 consisted of 62 patients. Nineteen (31%) patients were fully vaccinated; 16 (84%) of them had a mild and 3 (16%) a moderate clinical presentation. On average, the vaccinated patients contracted SARS-CoV-2 21.3 (S.D.=9.2) weeks after receiving the second dose of the BNT162b2 Comirnaty vaccine. Three of these 19 patients contracted the infection twice, first before vaccination (before April 2021) and for the second time after receiving both doses of vaccine. None of the patients were infected between the first and the second vaccination.

The other 43 (69%) patients were infected between April 2021 and April 2022 and were not vaccinated; three (7%) were asymptomatic, 30 (70%) had a mild and nine (21%) had a moderate clinical presentation, one (2%) required hospitalization. It seems that pARD who were not vaccinated before COVID-19 had a more challenging clinical presentation of the infection than those who received two doses of the vaccine before they were infected. The difference in the severity of clinical presentation of COVID-19 between vaccinated and unvaccinated pARD was not statistically significant (p = 0.31). The one patient that required hospitalization due to SARS-CoV-2 infection was unvaccinated.

We also compared the severity of the clinical presentation of COVID-19 between pARD who were receiving medication and those who were not on any medication at the time of infection. The results were not statistically significant (p = 0.31). Furthermore, we compared the severity of the clinical presentation of COVID-19 between pARD without medication and pARD who were receiving bDMARDs at the time of infection. Again, the results were not statistically significant (p = 0.68).

### Relapse rate of autoimmune rheumatic disease after COVID-19 and after vaccination against COVID-19

Of 103 infections with SARS-CoV-2, relapse of ARD was registered after 10 (10%) infections. The relapse was mild in four and moderate in five cases. One patient had a severe relapse of ARD and required hospitalization.

Among pARD who had a relapse after infection (Group 1), seven had JIA, one had systemic JIA (sJIA), and the other two had Sjögren’s syndrome and chronic idiopathic uveitis, respectively. One patient among the seven with JIA had a relapse of uveitis, so JADAS10 could not be evaluated. For the patients who had a relapse of ARD after COVID-19, the JADAS10 before and after the infection are represented in Table 3, where applicable.

**Table 3:** pARD with a relapse of ARD after infection and after vaccination with JADAS10 where applicable

Patient number	Diagnosis	Severity of relapse	JADAS10 (before)	JADAS10 (after)
Group 1 (after infection)				
1	JIA^*a*^	Mild^*c*^	0	3
2^* g*^	JIA	Mild	1	1
3	JIA	Mild	0	4
4	Sjögren’s syndrome	Mild	N/A^*f*^	N/A
5	JIA	Moderate^*d*^	3	5
6	JIA	Moderate	8	10
7	JIA	Moderate	3	19
8	JIA	Moderate	N/A	N/A
9	Chronic idiopathic uveitis	Moderate	N/A	N/A
10	sJIA^*b*^	Severe^*e*^	N/A	N/A
Group 2 (after vaccination)				
11	JIA	Mild	1	8
12	JIA	Mild	0	6
13	JIA	Moderate	6	18

^a^ JIA – juvenile idiopathic arthritis, ^b^ sJIA – systemic juvenile idiopathic arthritis, ^c^ Mild relapse – no treatment adjustment was required, ^d^ Moderate relapse – required a treatment adjustment, ^e^ Severe relapse – required a treatment adjustment in the hospital (hospitalization), ^f^ N/A – not applicable, ^g^ Patient had a transient worsening by description of the parents, on examination JADAS10 before and after was the same (problems subsided before we saw the patient for a check-up)

On average the before JADAS10 in Group 1 was evaluated 10.1 (S.D.=7.5) weeks before the infection, and the after JADAS10 7.2 (S.D.=4.4) weeks after the infection.

Patients in Group 1 who had a relapse of ARD after infection seem to have had a more challenging clinical presentation of the infection than those who did not have a relapse (Table 4). However, the difference in relapse rate depending on clinical presentation of COVID-19 was not statistically significant (p = 0.25).

**Table 4:** Clinical presentation of COVID-19 and relapse of ARD after infection

Clinical presentation of COVID-19	All infections	Relapse N (%)
Asymptomatic^*a*^	14	2 (14)
Mild^*b*^	69	4 (6)
Moderate^*c*^	19	3 (16)
Hospitalization^*d*^	1	1 (100)

^a^ Asymptomatic infection – no reported symptoms, ^b^ Mild clinical presentation – loss of smell and/or taste, fever of 38 °C or less for up to two days of, mild headache and/or cough with no other symptoms, ^c^ Moderate clinical presentation – fever lasting more than two days or above than 38 °C, myalgia, and/or other symptoms not included in the mild clinical presentation subcategory, ^d^ Hospitalization – because of COVID-19 patient required hospitalization

In Group 2, three (6%) among 47 vaccinated pARD had a relapse of ARD after vaccination. The relapse was mild in two pARD and moderate in one pARD. The patient who had a moderate relapse of disease after vaccination had an active ARD before vaccination with a JADAS10 score of 6. Severe relapses of ARD within this group were not recorded.

All three patients who had a relapse of ARD after vaccination had JIA. JADAS10 before and after vaccination against COVID-19 are presented in Table 3. On average the before JADAS10 in Group 2 was evaluated 5.7 (S.D.=1.2) weeks before the first dose of vaccine, and the after JADAS10 6.3 (S.D.=5.2) weeks after patients received the dose of the vaccine that caused the relapse. Two pARD had a relapse after the second dose and one after the first dose, however, he was still able to get the second dose as planned (4 weeks after the first dose).

The difference between the relapse rate in Group 1 (after infection) and Group 2 (after vaccination) was not statistically significant (p = 0.76). Moderate relapse of ARD was more common after the infection compared to vaccination, but the difference was not statistically significant (p = 0.19).

We looked at the occurrence of a relapse of ARD within three groups of patients: without medication, on bDMARDs, and on other medication, at the time of infection or vaccination. The results are presented in Table 5. No statistically significant differences were noted (p = 0.09 after infection and p = 0.30 after vaccination).

**Table 5:** Relapse of ARD after infection and vaccination based on medication of ARD

	No medication N (%)	On bDMARDs N (%)	On other medication N (%)
*Group 1 (after infection)*			
Relapse YES	0 (0)	8 (8)	2 (2)
Relapse NO	25 (24)	44 (43)	24 (23)
*Group 2 (after vaccination)*			
Relapse YES	0 (0)	3 (6)	0 (0)
Relapse NO	15 (32)	19 (41)	10 (21)

## Discussion

This prospective single-centre study focused on the clinical presentation of COVID-19 in pARD and the relapse rate of ARD in patients after COVID-19 infection and vaccination. The research was done amongst children, adolescents, and young adults with ARD. Currently, there are only a few studies reporting on the relapse rate of ARD after COVID-19 and/or after vaccination against COVID-19 [8, 11, 23]. Therefore, this study contributes to the enrichment of knowledge in this field.

### Clinical presentation of COVID-19

In our study, most patients were asymptomatic (14%) or had a mild clinical presentation (67%). Together, they accounted for 81% of all infections. A moderate clinical presentation was present in 18% of patients, and one patient (1%) was hospitalized. No-one suffered respiratory distress, and no-one required oxygen. The one patient who required hospitalization was admitted for dehydration. He had not been vaccinated prior to infection. None of the patients presented with MIS-C symptoms. Both patients treated with rituximab did not require hospitalization; one presented with a mild and the other with a moderate clinical presentation of COVID-19. In case of infection with delta virus they were advised to receive Regeneron, however, one notified us about the disease only after three weeks, and the other one had the omicron virus, so none received the antibodies. They also did not experience a relapse of rheumatic disease after COVID-19.

These findings are comparable to the available research. In a Turkish study by Sozeri et al., 113 children and adolescents with ARD on bDMARDs who recovered from COVID-19 were enrolled; 42 (37%) were asymptomatic, and 71 (63%) had COVID-19 symptoms. Of these 113 patients, 24 (21%) required hospitalization, and two (2%) even required intensive care therapy [24]. Another study done in the USA by Villacis-Nunez et al. included 55 children and adolescents with ARD after COVID-19 infection. Of the 55 patients, 10 (18%) were asymptomatic, 35 (64%) had a mild or moderate clinical presentation, and 10 (18%) were hospitalized [5].

In our study, asymptomatic clinical presentation (14%) was less common than reported in previously published studies. This was directly reflected in a higher number of patients with mild and moderate clinical presentation (85%). However, it was interesting to note, that only one patient (1%) required hospitalization, compared with 24 (21%) in the Turkish study, and 10 (18%) in the American study.

### Relapse rate of rheumatic disease

Regarding the main objective of this study, we recorded 10 (10%) relapses of ARD after infection (Group 1) and 3 (6%) relapses of ARD after vaccination (Group 2). We observed a more challenging clinical presentation of COVID-19 in patients from Group 1 who later had a relapse of ARD, however, the connection between the severity of clinical presentation of COVID-19 and the relapse rate of ARD was not statistically significant (p = 0.25).

It is important to note, that in Group 1, four (40%) relapses were mild, five (50%) were moderate, and one (10%) was severe, and the patient required hospitalization to stabilize her ARD. In Group 2, two (66%) relapses were mild, one (33%) was moderate, and no one had a severe relapse of ARD after vaccination. Furthermore, the patient who had a moderate relapse of ARD after vaccination had an active disease with a JADAS10 score of 6 before vaccination. Usually, we aim to vaccinate pARD when the disease is in remission [25]. However, because of the uncertainty surrounding COVID-19, there was a higher risk of infection, so pARD got vaccinated regardless of their current disease status.

We also observed that of the ten pARD who had a relapse after infection (Group 1), two patients received both doses of the vaccine before contracting COVID-19, and the other eight received none. It is important to note, that the two vaccinated patients had a relapse of the ARD twice. First, after they received the second dose of the BNT162b2 Comirnaty vaccine, and for the second time after COVID-19. Therefore, these two patients also represent two thirds of pARD who had a relapse after vaccination (Group 2). They had COVID-19 24.1 and 15.4 weeks, respectively, after second vaccination with BNT162b2 Comirnaty vaccine.

There was no statistically significant difference between relapse rates after infection and after vaccination (p = 0.75). However, there was a trend towards higher and more challenging relapse after the infection compared to vaccination.

Only a few studies have been published, reporting relapse rates of ARD in children and adolescents after COVID-19 or vaccination against COVID-19. A German study included 988 children with JIA and serology for COVID-19 was determined in 178 of them. Of these, 13 samples were positive. A relapse of ARD was observed in 7 out of the 13 children (54%). Two children had signs of arthritis flare after discontinuation of medication, and the remaining five had a relapse of ARD after COVID-19 even though the medications for their ARD remained unchanged [8]. We can conclude that the relapse rate of ARD after COVID-19 in our study (10%) is much lower compared to the German study (54%). In our study, one patient had a relapse after stopping the bDMARDs and the second one had a relapse of ARD after lowering the dose of steroids, both changing their medications during the infection. This is comparable to the available data because we know that discontinuation of medication for ARD during COVID-19 can often lead to the exacerbation of ARD [26].

A Turkish study by Haslak et al. enrolled 246 vaccinated adolescents with autoinflammatory and rheumatic diseases. They recorded a relapse in 27 (12%) patients [25]. An Israeli study by Henshin-Bekenstein included 91 adolescents with juvenile-onset autoimmune inflammatory rheumatic diseases. They reported changes in immunomodulatory drugs for eight (9%) patients after the first dose and for an additional four patients (5%) after the second dose of the BNT162b2 Comirnaty vaccine. Worsening of ARD symptoms was noted in five (6%) patients after the first dose and in one (1%) after the second dose, possibly demonstrating that not every medication change was a result of a disease relapse [11]. These results are mostly comparable with the relapse rate of ARD in our study (6%). It is important to note, that one of our patients with a relapse of ARD after vaccination was not in remission at the time of receiving the vaccine (JADAS10 score of 6), and the second one discontinued part of her ARD medications on her own. Therefore, we cannot say with certainty that the relapse occurred because of vaccination.

The strength of this study is a relatively large study sample compared to the other published studies. Additionally, to our knowledge, this is the first study comparing relapse rates of ARD in paediatric patients after COVID-19 and after vaccination against COVID-19. Furthermore, it is important to note, that pARD, who were vaccinated, received two doses of the vaccine, respectively, but we counted both events as one, because the time span between the two doses was too short for the evaluation of the disease relapse after every dose.

This study also has some limitations. First and foremost, we must acknowledge the age difference in the patients enrolled in the study. Patients in Group 1 (after infection) ranged from 2 to 23 years, and patients in Group 2 (after vaccination) ranged from 10 to 21 years, since most vaccinated patients were 12 years old or older. The study only included patients from University Children’s Hospital Ljubljana, meaning it is a single-centre experience.

## Conclusions

Relapses of ARD are possible after COVID-19 as well as after vaccination against COVID-19. This study shows a trend toward a higher relapse rate after infection compared to vaccination, but further studies are needed. It is important to note, even though it was not proven statistically significant, that patients who got COVID-19 after receiving two doses of the BNT162b2 Comirnaty vaccine had a milder and less demanding clinical presentation of the disease. Furthermore, we observed a tendency for more frequent relapses of ARD, when the patient had a more challenging clinical presentation of COVID-19. However, it must be noted, that the results were not statistically significant.

## Electronic supplementary material

Below is the link to the electronic supplementary material.

Supplementary Material 1

## Data Availability

The datasets used and/or analysed during the current study are available from the corresponding author on reasonable request.

## Abbreviations

ARD: Autoimmune rheumatic diseases
bDMARDs: Biologic disease-modifying anti-rheumatic drugs
CAPS: Cyopyrin-associated periodic syndrome
csDMARDs: conventional synthetic disease-modifying anti-rheumatic drugs
COVID-19: Coronavirus disease 2019
CRMO: chronic recurrent multifocal oseomyelitis
JADAS10: Juvenile arthritis disease activity score 10
jDM: juvenile dermatomyositis
JIA: Juvenile idiopathic arthritis
jLS: juvenile localized scleroderma
pARD: Paediatric patients with autoimmune rheumatic diseases
SARS-CoV-2: Severe acute respiratory syndrome coronavirus 2
sJIA: Systemic juvenile idiopathic arthritis
SLE: Systemic lupus erythematosus
UCTD: Undifferentiated connective tissue disease
VAS: Visual analogue scale
